# Supplementary material for: A PRISMA systematic review of adolescent gender dysphoria literature: 2) mental health
Source: PLOS Glob Public Health. 2022 May 4;2(5):e0000426. doi: 10.1371/journal.pgph.0000426 (PMC10021389; doi:10.1371/journal.pgph.0000426)
Supplement: Attachment — Submitted filename: Response to reviewers.docx [file pgph.0000426.s002.docx]

**THE SAHLGRENSKA ACADEMY**

**Institute of Neuroscience and Physiology**

**Gillberg Neuropsychiatry Centre**

**Kungsgatan 12**

**411 19 Göteborg**

Dear Runsen Chen

Re: A PRISMA systematic review of adolescent gender dysphoria literature: 2) mental health

We very much appreciate the timely and constructive comments from reviewers on our manuscript. We have addressed each of their points in turn in the table below. Please note we have also implemented some changes based on feedback on our first paper re. language (e.g., sex rather than gender). All documents have been uploaded in the appropriate format. We hope this will allow our paper to be published in the near future.

|  | **Reviewer comment** | **Authors’ response** |
| --- | --- | --- |
| 1.0 | This was a pleasure to read. The manner through which the information is presented in a gender-sensitive manner was much appreciated. More specifically, your observation that the majority of participants in previous work identified as gender binary and that other identities remain under-represented frames the paper exactly where it needs to be. | Many thanks for this positive feedback. We did work hard to place the literature in the modern and ever-evolving context. |
| 2.0 | This is a well-written, detailed paper outlining the findings of a systematic review on GD and mental health. It requires minor revisions to explain the eligibility and exclusion criteria. Some broader reflections on the state of the literature at the global level would also strengthen the paper. | Many thanks for your positive and constructive feedback. |
| 2.1 | In the Introduction, it would be helpful to situate the developments around GD within global health. I.e. in which settings are these debates and policies being established, and where is progress lagging? Can you provide broader context around how the issue is positioned at the global level? | We have developed our paragraph addressing the intense debate on the subject (lines 78-85) to ground this more firmly in terms of global public health. It is difficult to say more as the existing research is based largely in Europe and North America, and it is here where the current debate appears to be focused (and thus the motivation for conducting this review). The fact that this debate is intensifying in countries where gender fluidity has only recently become more socially acceptable is interesting in and of itself, but we consider that a fuller exposition of this issue within this paper would detract from the main focus. |
| 2.2 | On p. 3 (lines 69-71), there is a reference to ‘inherent bias’ in studies. Can you explain this further? | We have added some text to this section to illustrate the elements of bias we encountered. Specifically, Anderssen et al (2020) conducted a survey where gender identity was given, but natal sex not. The authors acknowledged this made it impossible for them to know if any respondents in the ‘cis gender’ group had a gender other than that assigned at birth. Connolly et al (2016) acknowledged the socioeconomic bias in samples in the current research, given that we are dependent on sampling from those presenting at treatment centres, a resource not available to all. |
| 2.3 | On p. 3, line 77, you reference ‘lack of good quality evidence’ – what evidence specifically is lacking? Do you mean evidence about the interaction between GD and mental health? | We have added a few words to this sentence to be specific. We are referring to the field more broadly at this stage, although the same applies to research on GD and mental health more specifically. De Vries et al (2021) refer to the acknowledged lack of evidence within the field in their assessment of the Bell vs Tavistock case, including the need for more long-term follow-up of participants. The SBU (2019) report refers also to the lack of long-term follow-up, as well as the predominance of observational studies and lack of randomised controlled trials. |
| 2.4 | On p. 3, line 82, the reference to ‘the second of three sets of questions’ is confusing especially when followed by the list of seven questions (not appearing in sets) where Q4 is in bold. The explanation in lines 93-95 make sense but the initial explanation before this is not clear. | We have attempted to make this clearer by revising the text. We are reluctant to deviate from the existing format too much as it will impact on how this paper relates to paper 1 (now published). |
| 2.5 | In your eligibility criteria you mention the huge amount of non-peer-reviewed literature. Can you say more about this? If not peer-reviewed, who is conducting/commissioning this body of literature? Was the grey literature primarily guidance materials or original research? Did the grey literature include research conducted in low and middle-income settings? Understanding the state of the non-peer reviewed literature is important in understanding why this literature was excluded. Could you please provide more justification to explain the decision to exclude grey lit especially given how lack of evidence is painted as a significant problem in the Introduction of the paper? This may need to be mentioned in the limitations. | There were a lot of conference proceedings appearing in the searches, suggesting a growing and evolving body of work that has not yet reached the stage of peer-reviewed journal publication. There were also a lot of guidelines, clinical reviews and commentary pieces, which would not be able to answer our research questions. The decision to exclude grey literature was partly practical (we did not have the resources to include it), but also because we were keen to review the literature that had reached the most highly recognised level of quality (albeit with awareness of publication bias always being a risk). We wished to see what the science published according to a particular scientific standard could say on the subject. This is not to say that grey literature does not always meet a certain scientific standard, but that this is less likely and not always easy to verify. We do acknowledge the potential strength of including grey literature and have added mention of this to limitations. |
| 2.6 | On p. 5, line 137, you state that epidemiological, clinical and survey data are original data. Did you exclude qualitative studies, and if so, why were these studies excluded? This needs to be stated clearly as it sounds like qualitative studies were excluded. This may need to be mentioned in the limitations section of the paper. | As above, there were a range of qualitative studies appearing in our searches, but these would not have helped robustly answer our research questions. Qualitative studies tend not to include total samples and are much more prone to subjective interpretation than some of the quantitative indicators we were interested in for this paper. We do acknowledge the significant contribution that qualitative work can make to our broader understanding in this field, and have added mention of this in limitations. |
| 2.7 | Was any software used in screening? | Yes – we used Endnote (vX7.8) to manage the screening process. We also referred to Bramer et al (2016) and Peters (2017) in de-duplicating and managing the data within Endnote. We have added this information to the Methods section. |
| 2.8 | p. 15, line 474 states that the quality of the literature needs to improve to understand the needs of individuals, but based on what has been stated in the paper, it is not simply about quality but also the quantity. There is an opportunity here to also draw attention to the geographical settings in which most research is occurring. | Thank you for this observation. We agree that quantity is also an issue and have added wording to this effect. We mention the geographical settings in lines 469-483 and agree that this literature is currently very restricted in that respect. |
| 3.1 | why the title has number 2? Is it necessary to have 2 mental health? If the other papers are not necessarily related to the current review, I think it is not necessary to mention 2. | This paper is the second in a series of three based around a single set of searches. It had originally been planned as a single paper, but through the process it became clear it was desirable to break down the findings into three distinct papers. They are related as they address a broader question of ‘what does the literature tell us about gender dysphoria in adolescence?’. The relatedness of the three papers is explained fully in the Methods section. Paper 1 has already been published (titled ‘A PRISMA systematic review of adolescent gender dysphoria literature: 1) epidemiology’) so consistency is preferable. |
| 3.2 | Mental health is a very broad area. It will be more helpful to have a specific area to review. | Yes, it is. We intended to include any evidence of mental health comorbidity with GD. Had we included only a specific subset of mental health conditions, we would have missed important information. |
| 3.3 | There are already existing reviews on mental health and GD. The authors mentioned they found "none". What are the specific researching strategies to find none? | We searched PROSPERO for any planned systematic reviews, and our searches for our review brought up no existing systematic reviews that could answer the research question we had set (specifically focused on mental health and GD in adolescence). For example, the review by Dhejne et al (2016) cited in our paper excluded those under age 18 and was not conducted / reported according to PRISMA standards. The more recent PRISMA systematic review by de Frietas et al (2020) also excluded adolescents, and that by Frew et al (2021) focused on children. Although our search strategy is detailed in the paper, we have added some of this detail about other reviews for the benefit of readers who may have the same question. |
| 3.4 | The introduction did not clearly present the importance of the study. For example, why specific focus the adolescents, compared to adults? | In the Introduction we refer to the sensitive time period of adolescence in terms of pubertal development and gender identity. We made the case that it is particularly important therefore that treatments that are irreversible be fully evidence based. We are not sure what else we could have done to make the case for focusing on adolescents. |
| 3.5 | In the method section, why chose 12-17 as adolescents? Need to list the criteria. | Adolescence is generally regarded as beginning around puberty / the onset of the teenage years (i.e., 12-13). People are deemed as having reached maturity, or adulthood, once they reach the age of 18, and puberty is likely to have commenced and significantly progressed by this stage. Our criteria are listed in full on pages 5 and 6. |
| 3.6 | It is necessary to list the study types, and "not review" is not enough. | Apologies – I am not sure which part of the manuscript this comment relates to. |
| 3.7 | The searching terms are not adequate. | It is not clear in what way the reviewer considers the terms ‘not adequate’. We deliberately applied broad search terms to ensure we captured as wide a literature as possible. We took the advice of an experienced systematic reviews librarian and have transparently reported our rationale (we have added wording on p5). |
| 3.8 | We will suggest the authors read more about how to conduct a systematic review and the standardised procedures. | Many thanks for this advice. Systematic review methodology is indeed very specific and it takes training and experience to do them well. I (lead author) first published a systematic review in 2009 and have published several since. The team on this paper includes two senior professors with extensive experience of evidence-based medicine and systematic reviews. We also took advice from a well-qualified and experienced systematic review librarian in conceiving and conducting the searches. We consider ourselves to have the appropriate level of training and experience. We have also pre-registered our review with PROSPERO and used the PRISMA 2020 statement to guide our review and its reporting. It is unclear what further action we might practicably take. |

We look forward to hearing from you.

Yours sincerely

Lucy Thompson (on behalf of the authors)
